# Altered blood parameters in “major depression” patients receiving repetitive transcranial magnetic stimulation (rTMS) therapy: a randomized case-control study

**DOI:** 10.1038/s41398-024-02942-8

**Published:** 2024-06-25

**Authors:** Beyza Nur Ozkan, Kubra Bozali, Muhammed Emin Boylu, Halil Aziz Velioglu, Selman Aktas, Ismet Kirpinar, Eray Metin Guler

**Affiliations:** 1 Department of Medical Biochemistry, University of Health Sciences Turkey, Hamidiye School of Medicine, Istanbul, Türkiye; 2 Department of Medical Biochemistry, University of Health Sciences Turkey, Hamidiye Institute of Health Sciences, Istanbul, Türkiye; 3 Department of Psychiatry, Bezmialem Vakif University, Faculty of Medicine, Istanbul, Türkiye; 4 Expertise Department of Psychiatric Observation, Council of Forensic Medicine, Ministry of Justice, Istanbul, Türkiye; 5 Center for Psychiatric Neuroscience, Feinstein Institute for Medical Research, Manhasset, NY USA; 6 Department of Neuroscience, Istanbul Medipol University, Istanbul, Türkiye; 7 Department of Biostatistics, University of Health Sciences Turkey, Hamidiye School of Medicine, Istanbul, Türkiye; 8 Department of Medical Biochemistry, University of Health Sciences Turkey, Hamidiye Faculty of Medicine, Haydarpasa Numune Health Application and Research Center, Istanbul, Türkiye

**Keywords:** Diagnostic markers, Prognostic markers, Depression

## Abstract

Major depressive disorder (MDD) is a debilitating illness that includes depressive mood. Repetitive Transcranial Magnetic Stimulation (rTMS) is a therapy method used in the treatment of MDD. The purpose of this study was to assess neurotrophic factors, and oxidative stress levels in MDD patients and evaluate the changes in these parameters as a result of rTMS therapy. Twenty-five patients with MDD and twenty-six healthy volunteers with the same demographic characteristics were included in the study. Brain-derived neurotrophic factors were measured photometrically with commercial kits. Oxidative stress parameters were measured by the photometric method. Oxidative stress index (OSI) and disulfide (DIS) levels were calculated with mathematical formulas. In this study, total antioxidant status (TAS), total thiol (TT), and native thiol (NT) antioxidant parameters and brain-derived neurotrophic factor (BDNF), glial cell line-derived neurotrophic factor (GDNF), and allopregnanolone (ALLO) levels were reduced in pre-rTMS with regard to the healthy control group; TOS, OSI, DIS, and S100 calcium-binding protein B (S100B) levels were increased statistically significantly (*p* < 0.01). Moreover, owing to TMS treatment; TAS, TT, NT, BDNF, GDNF, and ALLO levels were increased compared to pre-rTMS, while DIS, TOS, OSI, and S100B levels were decreased significantly (*p* < 0.01). The rTMS treatment reduces oxidative stress and restores thiol-disulfide balance in MDD patients. Additionally, rTMS modulates neurotrophic factors and neuroactive steroids, suggesting its potential as an antidepressant therapy. The changes in the biomarkers evaluated may help determine a more specific approach to treating MDD with rTMS therapy.

## Introduction

Depression is a common chronic disease that reduces a person’s ability to enjoy life, causing changes in physical health associated with usual mood swings and mental disorders. The disease is estimated to affect 5.0% of adults and 3.8% of the total population [1]. There are various types of depression heterogeneously, and major depression is one of them [2]. Major depressive disorder (MDD) is a debilitating illness characterized by at least one distinct depressive episode that includes depressive mood and vegetative symptoms such as marked changes in sleep, appetite, mood, interest, pleasure, and cognition lasting at least two weeks [3]. Cognitive behavioral therapy or antidepressant drugs are utilized in the first-line treatment of the disease in the clinic. In psychotherapy, both treatments can be used support separately or in combination [4].

Transcranial magnetic stimulation (TMS) is a non-invasive procedure that uses a magnetic field to stimulate specific brain areas. Repetitive TMS (rTMS) is a therapy efficient for inducing changes in brain activity that can last beyond the stimulation period [5]. TMS involves the generation of a magnetic field that passes through the skull and into the brain, where it induces a current in the targeted brain tissue. TMS is often used to treat depression, anxiety, and other mental health conditions [6]. It is thought that it may be put forward as a potential therapeutic strategy to improve treatment outcomes in drug-resistant MDD patients, which are characterized by dysregulation of neurotrophic factors, and increased oxidative stress, and do not respond to drug treatment. Central to this strategy is the targeted attenuation of oxidative stress pathways, particularly lipid peroxidation, as well as the modulation of activated immune-inflammatory cascades. It has been suggested that it may specifically target oxidative stress pathways, primarily lipid peroxidation, as well as activated immune-inflammatory pathways [7, 8]. This therapeutic method is cost-effective, easy to use, and safe that has Food and Drug Administration (FDA) - approved rTMS treatment protocols for the treatment of drug-resistant cases of major depression [9].

The left dorsolateral prefrontal cortex (DLPFC) has been extensively studied for its involvement in executive functions, including working memory, decision-making, and cognitive control, all of which are often impaired in various neuropsychiatric disorders. Its pivotal role in modulating mood and emotional responses also makes it a key region of interest in the treatment of depression and anxiety disorders. The application of rTMS to the DLPFC has been shown to modulate neural activity in this region, thereby offering therapeutic benefits for patients with depression, as evidenced by numerous clinical trials and meta-analyses [10, 11]. One of the primary advantages of targeting the DLPFC with rTMS is the non-invasive modulation of neural circuits involved in mood regulation and cognitive functions, offering a potential therapeutic intervention without the systemic side effects associated with pharmacotherapy. However, a potential disadvantage includes the variability in response among individuals, which may be attributed to differences in anatomical and functional connectivity of the DLPFC across patients [12]. Moreover, while the DLPFC is a logical target for conditions like depression, the specificity of rTMS effects may not be as beneficial for disorders where other brain regions play more central roles. This necessitates a tailored approach in selecting the stimulation target based on the disorder being treated and the individual patient’s neurobiological profile.

Neurotrophic factors are a class of protein-structured molecules that affect an array of neuronal functions. The effects of neurotrophic factors on the nervous system include neuronal growth, neurogenesis, differentiation, stress response, the pathophysiology of mood disorders, and the effect of antidepressants [13]. These factors also have critical roles in the pathophysiology of mood disorders, the effect of antidepressants, and the stress response [14]. Neuroactive steroids contain hormonal steroids and neurosteroids, which act on the nervous system that are synthesized locally by both nervous tissue (in neuronal and glial cells) and endocrine glands [15]. Changes in the levels of neuroactive steroids participate in the pathophysiology of clinical conditions related to depression, contribute to the therapeutic effects of antidepressants, affect the neurochemical response to stress, and modulate depression-related behaviors [16].

Oxidative stress and inflammation are hypotheses for the depression pathophysiology. Oxidative stress appears as a consequence of the excessive production of reactive oxygen species and the inability of antioxidant defenses to balance this increase. The inflammatory process begins, which occurs through the activation of proinflammatory pathways associated with oxidative stress [17, 18]. According to the neurotrophic and neurosteroid hypothesis; neuronal atrophy, neuroplasticity, and abnormal neurogenesis occur, decreasing in the areas related to mood and memory in the brain due to a change in the expression of these factors [19]. Neurotrophic factors and neuroactive steroids also play a stress-protective role by affecting the sequence of events caused by the generation of reactive oxygen species and apoptosis that contain protein structures [20]. The aim of this study is to determine how rTMS treatment affects the levels of these parameters by comparing the levels of neurotrophic factors and oxidative stress parameters in MDD patients pre- and post-rTMS with healthy volunteers.

## Materials and methods

### Study design

The patients in the study were selected from the people between the ages of 18–45 who applied to the Bezmialem Vakif University Faculty of Medicine, Policlinic of Mental Health and Diseases. According to the Diagnostic and Statistical Manual of Mental Disorders-5 (DSM-5), the presence of at least 5 physical and non-physical symptoms, including low mood and apathy; MDD was diagnosed in individuals whose symptoms were severe enough to interfere with daily social and occupational functions or cause significant distress and lasted for at least 2 weeks [21]. Twenty-five patients diagnosed with MDD according to DSM-5 criteria that had rTMS indications and twenty-six healthy volunteers with the same demographic characteristics were included in the study. All volunteers in the study signed an informed consent form.

The data obtained as a result of the study are stated in three different categories: pre-rTMS group (MDD patients who did not receive rTMS treatment), post-rTMS group (MDD patients who received rTMS treatment), and control group (healthy volunteers). Volunteers in pre- and post-rTMS groups represent the same individuals, and the relevant biochemical parameters were measured in the same individuals before and after rTMS treatment. The control group consisted of individuals defined as healthy volunteers who did not have chronic diseases, did not consume alcohol or tobacco, and avoided the use of any drugs or supplements that would affect the biochemical parameters measured during the study period. The inclusion criteria for the study consisted of individuals aged 18 to 45 who had provided signed informed consent, met the diagnostic criteria for major depression according to the DSM-5, were currently undergoing antidepressant therapy, and exhibited the absence of any concurrent neurological disorders other than MDD. Presence of any chronic medical/neurological disorder that requires treatment other than major depression disorders (e.g., epilepsy, diabetes, etc.), accompanying psychopathologies other than anxiety disorders (Generalized Anxiety Disorder, Separation Anxiety Disorder, Social Phobia, Panic Disorder, Agoraphobia), using any psychotropic medication, clinical mental retardation, and active suicidal ideation were defined as exclusion criteria. Moreover, STROBE guidelines were used to evaluate the quality of the study [22]. The ethics committee of this study was attained from the University of Health Sciences Turkey, Hamidiye Scientific Research Ethics Committee with decision 20/8 with registration number 22/432. Helsinki guidelines were followed for all experiments.

### Montgomery–Asberg Depression Rating Scale (MADRS)

The Montgomery–Åsberg Depression Rating Scale (MADRS) was used as a clinical rating scale for measuring the severity of MDD patients [23]. The scale consists of ten items, each of which is rated on a scale from 0 to 6, with higher scores indicating more severe symptoms. The items assess various aspects of depression, including mood, feelings of guilt, sleep disturbances, and physical symptoms such as fatigue or changes in appetite. The cumulative scores obtained from these items yield a total score, thereby delineating the severity of depression experienced by the individual, with the scale’s range spanning from 0 to 60. MADRS Cut-off scores: “Scores between 0–6 indicate no symptoms; 7–19 indicate mild depression; 20–34 indicate moderate depression; 35–60 indicate severe depression” [24].

### Repetitive Transcranial Magnetic Stimulation (rTMS) Therapy Protocol

Stimulation coordinates were determined before rTMS therapy was applied to the patients, such as in the FDA-approved protocols. TMS pulses over the left motor cortex and DLPFC were administered with an 8-coil (AirFilm® Coil (AFC) - Rapid Version), and The Magstim® Rapid² Stimulator. The TMS coil was placed between electrodes FC3 and C3, in the optimal position for eliciting motor-evoked potentials (MEPs) from the right abductor pollicis brevis. This localized the motor cortex. The optimum DLPFC location was found using the 5 cm rule [25]. The optimal motor cortex and DLPFC positions were marked to ensure consistent coil placement. The coil handle was pointed perpendicular to the presumed direction of the central sulcus, backward and 45° to the mid-sagittal line rTMS was administered with a total of 20 sessions of FDA-approved MDD treatment protocol, every weekday for a month. An application was made to the DLPFC at 120% of the motor threshold, with a frequency of 10 Hz, for a total of 37.5 min, 4 s of stimulation time and 26 s of latent time [11, 26].

### Sample collection

Basal standardization was achieved by taking blood samples at 8.00 am. Blood was drawn from the women in the follicular phase, as neuroactive steroids are affected by the menstrual cycle. Approximately 5 mL of blood was drawn from each patient pre- and post-rTMS in sterile biochemistry gel blood tubes with a clot activator. Collected blood was centrifuged to obtain serum for 10 min at 3000 × *g* and kept at −80 °C until experiments.

### Oxidative stress

According to the Erel method total antioxidant status (TAS) [27], total oxidant status (TOS) [28], and oxidative stress index (OSI) [29] were evaluated to determine the serum oxidative stress level.

### Dynamic Thiol/Disulfide Homeostasis

In order to evaluate thiol/disulfide homeostasis, according to the method determined by Erel and Neselioglu, serum total thiol (TT) and native thiol (NT) levels were measured, and the disulfide (DIS) levels were determined with the formula [30].

### Biochemical parameters

The biochemical parameters BDNF (Mybiosource-MBS824804), GDNF (Mybiosource - MBS355310), S100B (BT LAB - E3669Hu), and ALLO (AssayGenie - HUFI02220) were measured with ELISA kits photometrically.

### Statistical analyses

All data were analyzed by the SPSS version 25.0 program (IBM, Armonk, NY, USA). Statistical significant value was defined as *p* < 0.05. Parametric data were stated as mean ± standard deviation. Non-parametric data were stated as median (min-max). The difference between groups’ Independent Samples *t*-test was utilized. The difference between the two parameters in the groups was analyzed with the Mann–Whitney *U* test. The comparison of two normally distributed dependent groups was examined by paired sample *t*-test. A comparison of two dependent groups not normally distributed was examined with the paired sample Wilcoxon signed rank test. The relationship between variables was examined with the Spearman correlation test.

## Results

The groups were expressed in terms of age (31.84 ± 8.06) and gender (33 Female + 18 Male). See Table 1 for the demographic characteristics of the volunteers. Although there was no statistically significant difference between the groups in this study. The majority of the volunteers in the study are undergraduates (43.5%). Volunteers in the patient group had second attacks (51.7%) mostly; additionally had no previous TMS treatment and electroconvulsive therapy (ECT) (87%).

**Table 1:** Descriptive characteristics of the volunteers (Control: Healthy Control & Patient: pre-rTMS).

Variables		Control (*n* = 25) mean ± SD	Patient (*n* = 26) mean ± SD	*p*-value
Age *year*		31.28 ± 6.77	32.88 ± 9.43	0.491^a^
Sex *n (%)*	Female	15 (44.1%)	19 (55.9%)	
	Male	10 (55.6%)	8 (44.4%)	0.432^b^
Education *n (%)*	Primary	4 (50%)	4 (50%)	
	High school	7 (41.2%)	10 (58.8%)	0.867^c^
	Undergraduate	14 (51.9%)	13 (48.1%)	
Attack *n (%)*	None	25 (100%)	0 (0%)	
	First	0 (0%)	1 (100%)	
	Second	0 (0%)	16 (100%)	
	Third	0 (0%)	7 (100%)	<0.001^c^
	Fourth	0 (0%)	2 (100%)	
	Fifth	0 (0%)	1 (100%)	
Previous TMS *n (%)*	No	24 (49%)	25 (51%)	0.275^c^
	Yes	0 (0%)	2 (100%)	
Previous ECT *n (%)*	No	24 (48%)	26 (52%)	0.549^c^
	Yes	0 (0%)	1 (100%)	
MADRS		32.15 ± 6.25	1.71 ± 1.57	<0.001^a^

*TMS* Transcranial magnetic stimulation, *ECT* Electroconvulsive therapy, *MADRS* Montgomery–Asberg Depression Rating scale.

^a^İndependent Samples *T*-test.

^b^Chi-Squared test.

^c^Fisher Exact Test.

In our study, BDNF, GDNF, and also ALLO levels were lower in the pre-rTMS group than in the healthy control group (*p* < 0.01). ALLO, a neuroactive steroid, was found to be lower in the healthy control group compared to the patients see for details Table 2 (*p* < 0.01). The oxidative stress levels increased pre-rTMS in major depression patients, and the levels of antioxidant parameters were lower than the control group statistically significantly (*p* < 0.01).

**Table 2:** Comparison of neurotrophic factors and oxidative stress parameters with the healthy control and patient groups (Control: Healthy Control & Patient: pre-rTMS).

Variables		Control (*n* = 25)	Patient (*n* = 26)	*p*
BDNF *ng/mL*	mean ± SD	8.02 ± 1.11	3.68 ± 0.9	<0.01*^a^
S100B *ng/L*		100.76 ± 20.25	194.8 ± 22.47	<0.01*^a^
ALLO *pg/mL*		1195.26 ± 158.42	694.34 ± 81.54	<0.01*^a^
TAS *mmol ascorbate Eq./L*		0.99 ± 0.12	0.72 ± 0.11	<0.01*^a^
OSI *AU*		10.79 ± 3.01	20.5 ± 5.01	<0.01*^a^
NT *μM*		422.68 ± 46.72	275.13 ± 78.5	<0.01*^a^
DIS *μM*		51.68 ± 33.32	79.89 ± 43.19	<0.01*^a^
GDNF *pg/mL*	mean (min-max)	416.01 (385.75–618.91)	217.08 (154.75–250.03)	<0.01*^b^
TOS *μmol H*_*2*_*O*_*2*_ *Eq./L*		10.08 (6.8–16.53)	14.68 (10.43–18.48)	<0.01*^b^
TT *μM*		516.62 (446.21–635.02)	429.17 (371.24–582.69)	<0.01*^b^

*BDNF* Brain-Derived Neurotrophic Factor, *S100B* S100 calcium-binding protein B, *ALLO* Allopregnanolone, *TAS* Total Antioxidant Status, *OSI* Oxidative Stress Index, *NT* Native Thiol, *DIS* Disulfide, *GDNF* Glial Cell Line-Derived Neurotrophic Factor, *TOS* Total Oxidant Status, *TT* Total Thiol.

^a^Independent Samples *T*-test.

^b^Mann–Whitney *U* test (*p < 0.01 alongside of patient).

In addition, it is seen in Table 3 that oxidative stress decreased with treatment and antioxidant parameters increased in comparison with pre-rTMS (*p* < 0.01). BDNF, GDNF, and also ALLO levels were increased, while S100B and oxidative stress were decreased statistically significantly with rTMS treatment (*p* < 0.01).

**Table 3:** Determination of changes in neurotrophic factors and oxidative stress parameters before and after Repetitive Transcranial Magnetic Stimulation (rTMS) treatment in patients with major depressive disorder.

Variables		Pre-rTMS (*n* = 25)	Post-rTMS (*n* = 26)	*p*
BDNF *ng/mL*	mean ± SD	3.61 ± 0.86	4.49 ± 1.18	<0.01*^a^
S100B *ng/L*		194.76 ± 22.91	153.84 ± 20.78	<0.01*^a^
ALLO *pg/mL*		691.94 ± 82.18	738.60 ± 83.46	<0.01*^a^
TAS *mmol ascorbate Eq./L*		0.71 ± 0.11	0.87 ± 0.12	<0.01*^a^
NT *μM*		279.42 ± 76.76	351.35 ± 36.87	<0.01*^a^
DIS *μM*		79.88 ± 43.18	65.47 ± 40.71	0.115^a^
GDNF *pg/mL*	mean (mşin-max)	249.92 (154.75–618.91)	255.36 (247.27–319.61)	<0.01*^b^
TOS *μmol H*_*2*_*O*_*2*_ *Eq./L*		11.97 (6.8–18.48)	11.65 (8.81–16)	<0.01*^b^
TT *μM*		459.17 (371.24–635.02)	473.44 (407.85–679.41)	<0.01*^b^
OSI *AU*		14.16 (6.58–28.15)	13.25 (9.49–26.77)	<0.01*^b^

*BDNF* Brain-Derived Neurotrophic Factor, *S100B* S100 calcium-binding protein B, *ALLO* Allopregnanolone, *TAS* Total Antioxidant Status, *NT* Native Thiol, *DIS* Disulfide, *GDNF* Glial Cell Line-Derived Neurotrophic Factor, *TOS* Total Oxidant Status, *TT* Total Thiol, *OSI* Oxidative Stress Index.

^a^Paired Sample t-test.

^b^Wilcoxon signed rank test (*p < 0.01 alongside of pre-treatment).

## Discussion

TMS has been used as a potential brain stimulant since its invention. However, the effect mechanism of TMS is still unknown. Today, TMS is used as a therapeutic tool [31] in a wide spectrum ranging from psychiatric [32] to psychotic [33] and neurodegenerative [34] diseases. TMS has many beneficial effects on patients. For instance, after TMS treatment, there are improvements in cognitive and behavioral situations such as executive, memory, depression, and orientation functions in these diseases [35, 36]. However, we have no data other than certain unproven hypotheses on how this happens [37]. Therefore, changes in blood values are important parameters to improve our idea about the effect mechanism of TMS. In this study, we examined the changes in neuroprotective, antioxidative, and anticholinergic parameters through blood samples of MDD patients and aimed to evaluate the effect mechanism of TMS.

The overproduction of reactive oxygen species triggers pathological stages and eventually leads to increased permeability of the blood-brain barrier, morphological changes in the brain, and neuroinflammation [38]. Evidence suggests that oxidative stress may also be increased in several psychiatric disorders, including depression [39]. Increased production of reactive oxygen species decreased antioxidant system activity, and reduced ability of the repair pathways are closely related to mental and neurological disorders such as MDD [40, 41]. Another parameter examined in our study to evaluate the oxidative stress level in MDD patients was thiol-disulfide homeostasis. Thiol is a natural compound containing the sulfhydryl (-SH) group that is found in cells and has a critical position against oxidative stress. Thiol groups of amino acids are the primary target point of oxygen radicals. Oxygen radicals and thiol groups are oxidized to form reversible disulfide bonds [42]. Oxidative stress causes disulfide formation by oxidation of cysteine residues. These disulfide bonds formed thiol groups because of sustained dynamic thiol-disulfide homeostasis [30]. In a study by Baykan et al., it was shown that the dynamic thiol/disulfide balance in women with a diagnosis of major depression without treatment was in the direction of thiol formation, suggesting an antioxidative reaction [43]. Another study by Karaaslan et al. revealed that thiol levels were reduced throughout the first episode of depression in MDD patients [44]. When we compared the blood samples taken from volunteers who came to the outpatient clinic in our study before and after rTMS treatment, it was seen that the oxidative stress index decreased in the post-rTMS group.

Additionally, it was observed that thiol-disulfide homeostasis shifted towards DIS formation after rTMS. In the post-rTMS group, a decrease in DIS formation, and an increase in TT and NT as antioxidant levels were detected. All these findings indicated that oxidative stress increased in MDD patients who did not receive rTMS. Although there was a decrease in oxidative stress with rTMS treatment, it was observed that the treatment did not provide a complete recovery as in healthy people.

Neurotrophic factors, which affect an array of neuronal functions, are a class of protein-structured molecules such as BDNF, GDNF, and S100B [45]. BDNF is a growth factor from the neurotrophin family. Proinflammatory cytokines and neuroinflammation are important in inflammatory responses, neurogenesis, and neuroprotection in major depression [46]. Although studies are showing a decrease in BDNF levels in the analysis of post-mortem samples taken from depression patients, it was observed that the BDNF levels of depressed patients who did not receive antidepressant treatment were significantly reduced in the post-mortem examination of patients who died by suicide when compared to the group that received antidepressant treatment [47, 48]. Another study found that BDNF levels were increased in depressed patients with antidepressant treatment [49]. In a meta-analysis study examining BDNF levels in MDD patients, serum BDNF levels were also decreased in MDD patients in comparison with the healthy control group [50]. GDNF is another neurotrophin commonly found in the brain. It affects the differentiation and improvement of neurons and protects neurons and glial cells. It has been reported that GDNF levels were lower in mood disorder patients in comparison to the control group, and decreased as the degree of depression increased significantly [51]. GDNF has an important role for neurons other than dopaminergic neurons; It is involved in the maintenance of the functional properties of cellular elements of the blood-brain barrier and blood-nerve barrier, including pericyte and endothelial cells [52]. In their study, Lee et al. showed that serum GDNF levels were similar between MDD patients and healthy controls; it was reported that serum GDNF levels were dramatically low in patients with recurrent depressive episodes compared with the first episode regardless of treatment patients [53]. S100B protein, a biomarker more closely associated with astrocytes in the brain, is one of the Ca^2+^ binding protein group subgroups [54]. It has been shown that the S100B level is significantly increased in psychiatric diseases, traumatic brain injuries, cerebrovascular pathologies, and neurodegenerative diseases [55]. In a study by Maier et al., they emphasized that microinflammation in the brain promoted by depression-induced neuronal corruption causes the compensative releasing of S100B against the neurodegeneration process of the MDD [56]. In this study, as a result of comparing the patient and healthy control groups with regards to serum BDNF and GDNF levels in pre- and post-rTMS groups, it was observed that the BDNF levels were statistically significantly lower in the pre- and post-rTMS patient group in comparison with the healthy control. However, an increase in BDNF and GDNF levels were observed in the post-rTMS group when pre- and post-rTMS were compared. The alteration observed in neurotrophic factors among MDD patients after rTMS treatment signifies the modulation of mechanisms governing neuroplasticity and the viability and functionality of neuronal cells within the brain. This increase suggests that it may support the antidepressant effect of rTMS by contributing to increased neuroplasticity in the brain and improving the functions of nerve cells. Morover, it was observed that the S100B levels were statistically significantly lower in the post-rTMS group compared to the pre-rTMS group. However, an increase in S100B levels was sighted in the pre-rTMS compared with the healthy control. The decrease in S100B in MDD patients treated with rTMS may indicate that inflammatory and neurotoxic processes in the brain have decreased and the mechanisms related to the protection of nerve cells may have improved. It has been suggested that this reduction may reflect a potential protective effect that may contribute to the antidepressant effect of rTMS.

Although neuroactive steroids are transcriptional factors effective in regulating gene expression, these factors can also alter neuronal excitability through specific neurotransmitter receptors. ALLO is one of the neuroactive steroids [57]. ALLO is a positive allosteric modulator targeted to the GABAergic pathway. Nevertheless, it is associated with the expression of nuclear receptors, calcium channels, and other molecular pathways and also contributes to the antidepressant effect [58]. Studies have revealed that decreased ALLO levels in cerebrospinal fluid (CSF) or peripheral blood samples are associated with major depression, impulsive aggression, negative symptoms in schizophrenia, and anxiety disorders [57, 59]. In our study, ALLO levels were reduced in MDD patients in comparison with the healthy control group. It was observed that ALLO levels increased with the rTMS treatment compared to the pre-rTMS group. Considering the findings, it is evident that the augmentation of ALLO levels among individuals diagnosed with MDD, particularly in conjunction with rTMS treatment, significantly takes charge of the antidepressant efficacy of rTMS.

One notable strength of the study lies in its comprehensive assessment of biochemical parameters within the patient cohort, both pre- and post-rTMS completion. This approach facilitates direct comparison with a healthy control group, enhancing the robustness of the findings. However, the study is constrained by its limited sample size and the relatively small number of patients enrolled, thereby necessitating caution in generalizing the results to broader populations. This study was carried out on samples taken from peripheral blood. It is known that CSF allows more accurate interpretation of relevant parameters, especially in psychiatric and neurological diseases. In addition to this advantage, it is quite difficult to obtain CSF samples in clinical practice. However, it is also known that samples taken from peripheral blood can be useful when supported by clinical evidence such as scale and neuroimaging. All these reasons are limitations for this study evaluating rTMS treatment in MDD patients.

In our study, we purposed to compare the changes in neurotrophic factors, neuroactive steroids, and oxidative stress between the MDD patient and the healthy control groups, and to evaluate these parameters according to pre- and post-rTMS. Our results show that rTMS treatment contributes to the reduction of oxidative stress and the restoration of thiol-disulfide homeostasis in MDD patients. It has also been determined that rTMS modulates the levels of neurotrophic factors and neuroactive steroids and that these factors are associated with treatment effectiveness. These findings suggest that rTMS may exert antidepressant effects as a potential therapeutic strategy in the treatment of MDD, and targeting these mechanisms may be a promising approach to improving treatment responses. It has been thought that the study’s results and the changes in these biomarkers may help to determine a more specific approach to treating MDD with rTMS therapy.

## Data Availability

Data are available from the corresponding author upon request.
