# Correction: Altered blood parameters in “major depression” patients receiving repetitive transcranial magnetic stimulation (rTMS) therapy: a randomized case-control study

**DOI:** 10.1038/s41398-024-02999-5

**Published:** 2024-07-11

**Authors:** Beyza Nur Ozkan, Kubra Bozali, Muhammed Emin Boylu, Halil Aziz Velioglu, Selman Aktas, Ismet Kirpinar, Eray Metin Guler

**Affiliations:** 1 Department of Medical Biochemistry, University of Health Sciences Turkey, Hamidiye School of Medicine, Istanbul, Türkiye; 2 Department of Medical Biochemistry, University of Health Sciences Turkey, Hamidiye Institute of Health Sciences, Istanbul, Türkiye; 3 Department of Psychiatry, Bezmialem Vakif University, Faculty of Medicine, Istanbul, Türkiye; 4 Expertise Department of Psychiatric Observation, Council of Forensic Medicine, Ministry of Justice, Istanbul, Türkiye; 5 Center for Psychiatric Neuroscience, Feinstein Institute for Medical Research, Manhasset, NY USA; 6 Department of Neuroscience, Istanbul Medipol University, Istanbul, Türkiye; 7 Department of Biostatistics, University of Health Sciences Turkey, Hamidiye School of Medicine, Istanbul, Türkiye; 8 Department of Medical Biochemistry, University of Health Sciences Turkey, Hamidiye Faculty of Medicine, Haydarpasa Numune Health Application and Research Center, Istanbul, Türkiye

**Keywords:** Diagnostic markers, Prognostic markers, Depression

Correction to: *Translational Psychiatry* 10.1038/s41398-024-02942-8, published online 25 June 2024

In this article, the order that the authors appeared in the author list was incorrect.

The original article has been corrected.

